# Vocal communication across cultures: theoretical and methodological issues

**DOI:** 10.1098/rstb.2020.0387

**Published:** 2022-01-03

**Authors:** Gregory A. Bryant

**Affiliations:** Department of Communication, Center for Behavior, Evolution, and Culture, University of California, Los Angeles, 2225 Rolfe Hall, Los Angeles, CA 90095-1563, USA

**Keywords:** vocal communication, cross-cultural, voice modulation, evolution

## Abstract

The study of human vocal communication has been conducted primarily in Western, educated, industrialized, rich, democratic (WEIRD) societies. Recently, cross-cultural investigations in several domains of voice research have been expanding into more diverse populations. Theoretically, it is important to understand how universals and cultural variations interact in vocal production and perception, but cross-cultural voice research presents many methodological challenges. Experimental methods typically used in WEIRD societies are often not possible to implement in many populations such as rural, small-scale societies. Moreover, theoretical and methodological issues are often unnecessarily intertwined. Here, I focus on three areas of cross-cultural voice modulation research: (i) vocal signalling of formidability and dominance, (ii) vocal emotions, and (iii) production and perception of infant-directed speech. Research in these specific areas illustrates challenges that apply more generally across the human behavioural sciences but also reveals promise as we develop our understanding of the evolution of human communication.

This article is part of the theme issue ‘Voice modulation: from origin and mechanism to social impact (Part II)’.

## 1. Introduction

Research on vocal communication is burgeoning, but few areas have grown as dramatically as cross-cultural investigations. In particular, voice researchers are beginning to extend their empirical reach into small-scale societies, and access populations that can potentially enhance our understanding of the complex relationship between universal patterns and cultural variations in vocal production and perception. Like most experimental social science, the study of vocal communication has been traditionally done using participants from the global North. But in the last decade or so, the situation has started to change. This represents an important turn in the scientific study of voices, but at every turn, there are new challenges. The cross-cultural study of vocal communication raises important theoretical and methodological issues, some long recognized, but others that are new and deserve our attention. In this review, I aim to describe some important difficulties facing cross-cultural voice researchers, and provide possible solutions moving forward.

The study of vocal communication is deeply interdisciplinary for fairly obvious reasons. Much of the theoretical work that informs current research comes from behavioural biology and animal signalling. Human vocal production and voice acoustics have been investigated by psychologists, linguists, neuroanatomists, engineers, and medical clinicians, among others. The underlying mechanisms of voice production are beginning to be mapped out, but our understanding of the communicative functions of vocalizations is much less developed. A major reason for this difficulty is that human vocal communication occurs in the context of complex social interaction, and importantly, interfaces directly with language. But much of human vocal behaviour is also non-verbal, including laughter, crying, moans, screams and roars. Non-verbal vocalizations have been traditionally understudied, but recent attention is changing that, including large-scale cross-cultural studies.

An important distinction for human vocal behaviour is that between spontaneous and volitional production [1,2], with most spontaneous production comprising non-linguistic vocalizations, and volitional production being speech and modulated forms of spontaneous non-verbal expressions [3]. Cross-species comparative approaches are important but also somewhat limited in helping us understand the unique features of human vocal modulation. That is, our ability to volitionally control our vocal production is species-specific, so it is currently unclear what insights can be garnered by examining volitional vocal control in non-human animals (but see [4]). Human communication generally manifests itself in conversation, a multimodal interactive phenomenon rooted in language. Voice modulation must be understood largely in this milieu, presenting researchers with a variety of empirical challenges. We must reverse-engineer design features of cognitive and behavioural mechanisms operating in the service of deeply contextualized conversational interaction, but most methods attempt to access these systems outside of this crucial context (e.g. through decontextualized experimental paradigms). Moreover, the problem of studying communication phenomena independent from typical interactive contexts is exacerbated by additional difficulties of examining human behaviour across cultures. Most people around the world have little or no experience with social science research, making experimental tasks additionally distanced from most phenomena of interest (e.g. how people interpret an isolated vocal recording as a proxy for interpersonal judgement processes). Vocal communication research, however, has one advantage over many areas of behavioural research: we can measure the physical properties of voices in objective ways, solving problems of equivalence that limit other kinds of cross-cultural investigators. A growing body of voice research reveals some very promising lines of inquiry.

A complete account of how cross-cultural research has shaped what we know about human vocal communication is obviously beyond the scope of one article. But here, I will focus on three broad domains of research on voice modulation. Currently, there are flourishing cross-cultural research programmes examining how people (i) modulate their voices in ways that signal dominance and formidability, (ii) generate emotional vocal signals for the navigation of social interactions, and (iii) communicate vocally with preverbal infants and young language learners. Each of these domains of vocal signalling has deep phylogenetic roots, but is also critically influenced by volitional voice modulation, a particularly human capacity [1,2]. Cross-cultural studies are illuminating the species-specific mechanisms and developmental trajectories of human vocal behaviour, helping us better understand their evolutionary histories and communicative functions.

## 2. Cross-cultural concerns

Several interdisciplinary scholars have recently described issues facing current cross-cultural researchers (e.g. [5–17]). Most of the concerns raised apply to vocal research in one way or another. But in the three general areas of work described here, I will point to two particularly relevant and overlapping issues for voice researchers: (i) conceptualizing the theoretical problems associated with measuring universals and cultural variation, and (ii) study design, including task demands and response variables.

A basic theoretical issue for cross-cultural research, in general, is the question of how to conceptualize and study universality and cultural variation. In the case of voices, how can we look for potential universals in vocal signals and cues,^1^ empirically? An important feature of signalling systems is accuracy: while there is always noise in any information channel, for a signal to evolve, there must be some degree of non-ambiguity in what the signaller intends, and in what the receiver derives from the signal. Intentions, of course, are not necessarily consciously accessible to signallers and receivers, but rather, are part of the design of the system. Thus, in tasks intended to measure the accuracy of various judgements of vocal stimuli across cultures or individuals, it is important that the stimuli and the response task be designed so that there is a correct answer. ‘Correct’ here does not mean correct in the opinion of the experimenter or according to some theory. Rather, it means that (i) participants who properly understand the task and comply will not vary in what they are trying to do, and (ii) there is an objective, observer-free, theory-free criterion for correct judgement (i.e. an accurate response is not dependent on choices by the experimenter). I will return to these issues.

When engaged in behavioural research with individuals from varying cultures who speak different languages, we face a significant challenge in ensuring that our participants understand what is being asked of them, and it is not as simple as merely developing direct translations of our instructions and materials. Peña [16] described four interrelated dimensions of equivalence in challenges for translation. Linguistic equivalence involves ordinary notions of translation that all cross-cultural researchers have completed: we get native speakers to translate relevant material, which is then translated back to the first language by a different speaker, and these are compared and adjusted if needed. It is the minimum that must be done to create potentially useable instructions and instruments across language groups. But this does not necessarily ensure functional equivalence, which is the guarantee that the translation will elicit comparable behaviour across participants. For example, rather than relying on word-for-word translation, materials sometimes should be created across different languages simultaneously with considerations of communicative pragmatics that might require altering word choices and phrasing. Relatedly, cultural equivalence requires sensitivity to possible differences in how certain concepts are interpreted even though they have been appropriately translated technically. Finally, metric equivalence refers to potential differences in the difficulty of understanding particular tasks or instructions, even controlling for functional and cultural equivalence. Without taking great care in ensuring these different aspects of equivalence, research findings can be at best noisy, and at worst biased, just owing to the construction of research materials across cultural and language groups.

The recent impetus for cross-cultural research in all areas of cognitive science, including voice production and perception, is due primarily to wide recognition of what scholars have identified as the Western, educated, industrialized, rich, democratic (WEIRD) problem. WEIRD participants constitute the bulk of research subjects in most studies in the behavioural sciences. Henrich *et al*. [11], who coined the WEIRD acronym, described several classic findings in perception, many widely considered universal psychological abilities, as often being subject to more cultural variation than previously believed. Regardless of the status of any given claim (many detailed debates exist regarding these issues), their point stands as critical: the behavioural sciences have been relying on one rather narrow demographic in the quest for understanding the nature of human cognition and behaviour. One popular response to this issue, with evolutionary behavioural scientists leading the way, has been to expand research into populations that are thought to differ radically from WEIRD people, often focusing on subsistence-based, small-scale societies.

Many researchers have used this approach as a strategy to provide the best possible evidence for the universality of a given behavioural trait, but also possibly for getting a better glimpse into the evolutionary nature of that phenomenon, treating some small-scale participants as proxies for our hunter–gatherer ancestors. This second assumption is especially problematic, however, and should be approached with caution. Barrett [7] called this the *ancestral gambit*—a tentative supposition that certain contemporary people live in ways that most resemble humans in our ancestral past, often without considering the complex historical factors that have led to various lifeways in these small-scale societies. Moreover, Barrett [7] argued that while studying groups residing in remote locations, and who have relatively less contact with WEIRD culture, can be valuable, a high proportion of the world's people are actually somewhere in between these extremes, and are relatively ignored by behavioural scientists. Researchers should take more care in choosing their study populations in ways that fit their research questions rather than opting for the most ‘traditional’ or isolated group they can find for the effect of presenting data from an exotic locale (see [8,9]). Human variation is present on many levels and across many domains—understanding that variation requires more than just close examinations of the continuum endpoints.

These concerns apply to all voice modulation research in various ways. In what follows, I will review three areas of cross-cultural investigation that represent current active domains of vocal communication research, and in each section, I will describe some of the theoretical and methodological issues that I believe present challenges researchers should address moving forward.

## 3. Vocal signalling of formidability and dominance

A growing body of cross-cultural research exists regarding the relationship between vocal acoustic features and the physical characteristics of speakers. Much of this work has focused on voice fundamental frequency (*f*_o_)—the vibration rate of the vocal folds—and to a lesser extent, formant frequencies (*F_n_*), which are the resonant properties of the vocal tract that manifest acoustically as clusters of energy that correspond to vocal tract structure and configuration [19]. The documented perceptual effects of vocal pitch (the perceptual correlate of *f*_o_) are vast and are beyond the scope of this review, but some relevant themes have emerged. One robust finding is that relatively lowered pitch is associated with ratings of dominance and strength, and these effects extend into many social judgements, from political prowess to sexual attraction (for reviews, see [20,21]). Most of this work is with WEIRD samples, but there are some exceptions. For example, in one study, Tsimané listeners (Bolivian forager–horticulturalists) rated pitch-manipulated spoken passages produced by US college men [22]. Lowered-pitch voices were judged by men, but not women, as being produced by individuals with greater fighting ability. Women judged the higher-pitched versions as being more attractive as potential mates. Interestingly, lowered *f*_o_ was not associated with judgements of prestige, a category created by asking about the respect, talent, success and admiration of the individuals they heard. Listeners seemed to restrict their pitch-related judgements to the physical attributes of the speakers. These findings were interpreted as supporting the theory that low pitch in males is the product of sexual selection.

The problem is that evidence for a relationship between actual strength and voice pitch is equivocal, and if true, the effects are small [20,23]. Sell *et al*. [24] found that American judges could infer strength from ordinary speech produced by Romanian college students, Argentinian herder–horticulturalists, Bolivian forager–horticulturalists and American students, but neither *f*_o_ nor *F_n_* was related to actual strength. Listeners used vocal pitch in their judgements, and got the right answer (i.e. rated stronger men higher than weaker men, on average) despite relying, in part, on that invalid cue. Of course, they must have tracked different acoustic features for accuracy. Other studies have reported acoustic correlates of physical strength. Puts *et al*. [25] found that in Hadza men (hunter–gatherers in Tanzania), individuals with greater arm strength, measured using a hand dynamometer, had lower *f*_o_—and in an American college student sample, lower formant position (*P*_f_) was associated with greater arm strength. In a study of Tsimané peripubertal males and females, actual strength in young males predicted *f*_o_ and *P*_f_ after controlling for height, body fat and age [26]. Other work in the same population [27] found that adolescent males' condition, operationalized as secretory IgA measures of immune response and adjusted BMI, was negatively associated with testosterone levels and lower *f*_o_ and *F_n_*_,_ supporting a costly signalling model of voice pitch. A recent meta-analysis indicated there is likely some relationship between *f*_o_ and formidability [20], but the modest relationship seems inadequate to fully explain the substantial perceptual effects of pitch on judgements of dominance and strength.

Feinberg *et al*. [28] presented a sensory exploitation hypothesis, arguing that pre-existing biases that associate low frequencies with large objects predispose judges to identify low-pitched voices as belonging to bigger, stronger speakers (see also [29]). There is evidence that people across cultures intuitively understand this and modulate their voices accordingly. For example, Pisanski *et al*. [1,2], instructed speakers from Canada, Cuba and Poland to alter their voices to sound either bigger or smaller, and these voice modulations were compared with their baseline vocal properties while producing vowel sounds. As expected, speakers from all three groups changed their voices similarly. When trying to sound larger, speakers adjusted their apparent vocal tract length (VTL) to seem longer (by lowering *F_n_*), while simultaneously lowering their *f*_o_. Speakers adjusted both dimensions in the opposite direction to sound smaller. In all three groups, men modulated their voices to a greater extent than women, and all speakers tended to rely differentially on *f*_o_ modulation.

If speakers’ voices are related to their body morphology, we might expect other relationships to be present as well, such as how particular vocal indicators of formidability are related to mating and reproduction. A number of cross-cultural studies have examined the relationship between reproductive success and vocal *f*_o_, but the effects are confounded. Apicella & Feinberg [30] found a negative relationship between Hadza men's *f*_o_ and the number of purported offspring, and in subsequent work reported that Hadza men and women judged opposite-sex individuals with lower *f*_o_ as better foragers (i.e. hunting and gathering). But women who were currently breastfeeding preferred men with higher *f*_o_ as potential mates and women who were not breastfeeding preferred men with lower *f*_o_. To make matters more complicated, a later analysis revealed that the relationship just described above between the number of offspring and men's *f*_o_ failed to hold when controlling for hunting reputation, and instead hunting reputation predicted reproductive success [31]. Another study explored the connection between *f*_o_ and reproductive success in the Himba, seminomadic cattle herders from northern Namibia [32]. In this analysis, *f*_o_ was not related to any reproductive variables in men, but instead higher *f*_o_ in women was associated with greater handgrip strength and number of genetic descendants (i.e. offspring and grandchildren). Taken together, these results are rather difficult to interpret confidently, but they point to possible connections between hormone-driven signals in vocal characteristics and reproductive outcomes, as well as perceptual effects in social decision processes such as choosing a mate and assessing a possible rival. Small, mediated effects indicate a complex picture that requires much more research to properly assess, including accessing larger participant samples for adequate statistical power.

One important feature of this area of research is that many of the variables researchers typically examine are objective measures, including acoustic features of voices, hormonal profiles and clearly defined dimensions of bodily characteristics such as strength and size. Complications arise, however, when these measures are integrated with subjective judgements in often artificial decision tasks such as asking people to judge unseen individuals for attractiveness or social status. In small-scale societies (and to some extent in WEIRD societies), the idea of judging people based on such limited information likely seems arbitrary and artificial. Perceptual experiments, of course, necessarily involve repeated exposures to multiple stimuli, many sounding quite similar even to trained ears. I believe it is safe to assume that naive participants can easily become confused, and consequently might gauge their answers to some extent according to subtle cues exhibited by researchers, who in many cases, must manually enter answers for them (e.g. participants who have no familiarity with a computer or are non-literate). For instance, repeated exposures may implicitly suggest to some listeners that they should be looking for objective differences across items when they do not exist.

The cognitive processes that underlie response patterns in such experimental contexts are inevitably going to be driven, at least in part, by pragmatic reasoning mechanisms responding to demand characteristics in the studies and settings. Participants in small-scale societies (and other populations as well) often understand that they are being compared with people from other societies, and they can easily appraise experimental tasks as intelligence tests, assuming that there is a right answer, when in fact there often is not one. Care should be taken in developing instructions to manage the trade-off between risks associated with participants being threatened by a presumed intelligence test or direct cultural comparisons, and instructions that elicit accurate responses while communicating to the subject that they are not being judged for their pattern of responses. Participants also realize, usually implicitly (like experienced WEIRD participants), that there are expectations of the researchers for a specific pattern of responses.

Judgements involving measures such as attractiveness, prestige, likability and so on are likely to elicit highly variable responses across cultures as a function of the vast possible influences on performance (e.g. willingness to conform to expectation, variations in the ability to assess researchers' goals, etc.). Conversely, judgements of objective criteria, such as strength and identity (e.g. identify which pictured person produced a vocalization), will afford greater uniformity as accuracy in the task will often be determined by abilities present in judges due to cognitive skills shared by all people worldwide (i.e. perceptual adaptations). Consequently, accuracy in a task with a technically correct answer often becomes a superior benchmark (compared with opinion judgements) by which we should judge universals and cultural variation in cognitive and perceptual performance. Possible exceptions to this would be cases where perception is shaped to be biased for reasons of error management [33]. For example, variations across participants with experience in predation threats could result in systematic biases towards over-perceiving certain predators in noisy stimuli because that kind of error is less costly than the inverse in a real-world environment. But that bias can only be gauged by a clear operationalization of what counts as correct. In the next section on vocal emotion communication, the issue of accuracy might be the most important single element in what otherwise appears to be a highly variable behavioural phenomenon.

## 4. Vocal emotions

Since Ekman's classic studies of universal patterns of emotional face expressions [34], researchers have attempted to identify properties of emotional expression that transcend cultural boundaries, and which aspects seem subject to important variation. Research on vocal emotions (i.e. the expression of emotions in linguistic and non-linguistic vocalizations) got a slightly later start than facial expressions, but there now exists a substantial cross-cultural literature focusing primarily on the ability of perceivers to detect emotion categories in verbal and non-verbal vocalizations. Theoretically, there are reasons to expect both variation and universals in vocal emotional signalling. Human languages and practices vary enormously across cultures, but in order to be evolutionarily stable, signalling systems must allow some means of resolving ambiguity between senders and receivers [18,35]. Emotional signalling systems in humans must have at least some universal functional properties that dovetail with cultural diversity rather than working against it. If communication systems are at least partly mediated through culturally evolved traits, such as language, then cultural variation should be expected. ‘Universality’, in this case, might manifest at higher organizational levels of the signalling system, rather than in carbon-copy identicality of particular signalling tokens across individuals or cultures. For instance, vocalizations conveying anger likely occur universally in all languages and cultures, but particular acoustic manifestations of anger could vary as a function of many linguistic and articulatory production factors, as well as pragmatic rules [36]. Such variation might appear to work against universality since the universals are not as well represented in the tokens. Language provides a good analogous example. Language learning mechanisms exist at a high level of linguistic organization, and as such manifest universally across all people, leading to variations in syntactic, lexical and phonological structure shaped by different language environments [37]. In general, a single developmental process can generate highly variable developmental outcomes as a function of cultural differences [38].

Minimally, we should expect consistent patterns in acoustic configurations in vocalizations to the extent that form–function relationships are present in the signalling system [39–41]. For instance, emotional experiences associated with high arousal and negative valence (e.g. fear) should have similar effects on vocal physiology regardless of the cultural background of the speaker or the language they speak, and these effects co-evolved with perceptual systems designed to process them. But culturally evolved pragmatic rules of how particular expressions, such as a fear scream, occur in social interaction will generate variation across cultural and linguistic groups. We might expect greater universality in vocal signals than in facial signals because vocalizations can have relatively more direct effects on receivers (e.g. loud noises inducing the startle reflex). The power of the voice to express emotion goes beyond simple mappings of form and function, however—recent work has demonstrated that many fine-grained categories can be produced and recognized [42], though confusion matrices reveal variation in people's judgements. One possibility is that extremely subtle form–function connections between vocal sounds and affect terms, intensified through cultural attractor dynamics [43], can drive noteworthy agreement through conventionalization. For example, Perlman *et al*. [44] showed in a laboratory-based vocal charades game how sound iconicity can drive the evolution of conventionalized vocal expressions (see also [45]).

Like most areas of cross-cultural research, early examinations of vocal emotions focused almost exclusively on WEIRD participants. A recent meta-analysis of this work examined 37 studies with vocalizations coming from 26 different cultural groups and perceivers from 44 cultures (defined by either country or language group), very few of which came from non-WEIRD societies [46]. Over two dozen emotion categories were included overall. The analysis confirmed an in-group advantage for emotion recognition, meaning that perceivers were significantly more accurate in identifying emotion categories from vocalizers in their own culture. Additionally, measures of cultural distance between vocalizers and receivers were negatively correlated—the further away listeners were culturally from the target vocal producers, the less accurate they were in identifying emotion categories.

These data were presented as evidence in support of the dialect theory of emotion communication, which conceptualizes emotions as manifesting in ways similar to linguistic dialects [47]. That is, over time, subtle stylistic idiosyncrasies emerge in emotional signal production within an interacting group, resulting in perceptible structural variations that impact non-verbal detectability across groups. As distance increases between groups, so do the magnitude and effects of these emergent differences. With language, as mutual intelligibility is reduced, diverging dialects must be identified as distinct languages. In the case of emotional expression, distinctions clearly have an upper limit (e.g. frowns will never likely function as smiles), though the extent to which affective expressions can become culturally unique is not well understood. Work on vocal emotions has focused intensely on perception across groups, with almost no work closely examining actual production distinctions across cultures in terms of vocal acoustics, and production mode. For example, volitionally produced vocal emotions might be more difficult to identify across distant cultures than their spontaneous counterparts, given the greater involvement of language-specific speech processes [1,39].

Given the complex relationship between emotion categories, expressive signalling and culture, the in-group advantage finding is not particularly surprising. One basic implication of this recent analysis [46] is that vocal emotions are clearly recognized across different cultures, and cultural variation also plays an important role. The pattern of data supports the idea that universals can be thought of as existing on a continuum, where particular systematic patterns allow wide recognition of many kinds of expression, and variations exist, attributable to what is likely a large set of social and biological factors.

An important set of methodological issues confuse our understanding of vocal emotions across cultures, traceable to the earliest studies of emotion recognition in faces (e.g. [37]). How do we test for universals? In a recent series of studies with Himba participants (Namibia), researchers with different theoretical perspectives, using very similar tasks and stimuli, each claim to find support for their respective views. In one of the earliest examinations of vocal emotion recognition in a small-scale society, Sauter *et al*. [48] found evidence for bi-directional recognition of non-verbal emotional vocalizations between Himba and British participants. The choice-from-array task used was relatively straightforward: short pre-recorded vignettes were presented to participants that described a situation where an individual experiences a specific emotion. Listeners were first asked to confirm the target emotion in the story, and all were able to do so eventually, with repeated telling if needed. They were then presented two non-verbal vocalizations, one representing the target emotion and one a distractor. Listeners then had to select the vocalization that matched the emotion portrayed in the vignette. For example, an individual will hear a story about a person who encounters a dangerous predator and feels scared, and they are then presented recordings of a fear scream and a cry, for instance. A ‘correct’ response, in this case, would be the selection of the fear scream.

Himba judges were able to identify the correct (i.e. intended) vocalizations produced by British speakers in several categories, including purported basic emotions such as anger, happiness, fear, disgust and sadness. British listeners were also able to recognize emotional portrayals by Himba speakers. This work was presented as evidence of cross-cultural universals of basic emotion categories. In follow-up work with the same population, a different research group failed to replicate the findings, and instead reported that participants only successfully recognized the intended emotion categories when the target vocalization and the distractor differed in valence [49]. Recognition was better than chance only when the distractor matched the target in arousal and not in valence, but not when the distractor differed from the target in both arousal and valence. A re-analysis of the data of Sauter *et al*. showed that this was not true in the original study [50]. Moreover, Gendron *et al*. [49] performed a free-labelling task in which participants were asked to provide verbal labels for the emotions portrayed in the recorded voices, and, not surprisingly, there was very little consistency in their answers. There are many degrees of freedom in how participants might respond in a free-labelling task (e.g. variations in task interpretation, issues with different forms of translation equivalence, etc.), making these data extremely difficult to interpret. Yet, the findings were provided as evidence against universality in emotion expression, and instead argued to support the authors' constructionist perspective, including the notion that participants can learn emotion categories from the experience of the forced-choice task.

Using the same method as Sauter *et al*. [48], Gendron *et al*. [49] did not confirm participants’ understanding of the emotion content in the presented vignettes, and as a result likely included participants who did not comprehend at least some proportion of the presented stories. These participants, therefore, were answering according to either a mistaken understanding, an incomplete understanding or random guessing. Because of this, the findings cannot provide evidence against universality—if participants do not understand the task completely, they are not generating interpretable data, even if their emotion concepts are culturally variable and constructed as the researchers believe. There is a difference between not having an emotion concept activated in a participant, and not establishing functional and cultural equivalence in the materials used.

Can the choice-from-array experimental paradigm teach naive participants emotion categories on the fly? By confirming participants' comprehension of the vignettes, including repeating an emotion vignette when necessary to ensure understanding, and exposing listeners to multiple trials containing a vocal exemplar of the intended category, participants could plausibly acquire rudimentary emotion concepts that they previously did not have [49,51]. A large developmental literature provides clear evidence that children use fast mapping in acquiring concepts with no training [52], though evidence for this in adults is equivocal [53]. Nevertheless, performance might exceed chance in the judgement task for categories they did not otherwise know. Of course, this view does not explain why participants’ judgements in Sauter *et al*. [48] were at chance in several conditions, aside from the possibility that some categories are easier than others to acquire. One direct prediction of a category learning effect is that performance should improve over time within a single study (i.e. increased selection of the intended target vocalization), but to my knowledge, this has not been tested.

Related work by the same team sought to explore the possibility of concept acquisition through the experience in a choice-from-array experiment [51]. This study included a different small-scale population (Hadza), as well as participants from the USA and China. The researchers identified emotion concepts not translatable to a single word in any of the languages of the groups studied, and created a set of vocalizations to represent the categories. For example, one concept (‘gigil’) is the ‘overwhelming urge to squeeze or pinch something that is very cute’ [51, p. 5], with vocalizations that contained positive-sounding, high-pitched squeals (e.g. ‘eeee!’). As predicted, participants across all three cultures were able to recognize several of the categories better than chance. By examining three cultural groups who had no language-based emotion concepts that mapped cleanly to newly presented concepts, the researchers intended to demonstrate that experience in a repeated-measures, choice-from-array task could generate data that would pattern similarly to earlier data purporting support for universality in emotions (e.g. [48]). Because the researchers created the vocalizations for the project, and the novel emotion concepts were at best only understood conceptually without a specific verbal label, they could not be universally recognized owing to innate emotion detection abilities or universals in the vocalization patterns. Or so the argument goes.

So how did these participants successfully perform in the task? One possibility is that people do acquire some basic understanding of a category upon exposure. It is also true, as many scholars have argued previously, that choice-from-array tasks can enhance performance on various kinds of detection tasks and inflate appearances of consistency across cultures (e.g. [54]). But even more importantly, the extent to which the listeners in this recent work performed successfully is likely due, in large part, to form–function relationships between the described emotion category and the intended target vocalizations. For example, the ‘gigil’ category described earlier is clearly positive—the presented story actually uses the words ‘strong positive feeling’ and the associated vocalizations were judged by a different set of listeners as positively valenced and high arousal. While arbitrary pairings are in principle learnable, prepared learning might make certain pairings more likely to be acquired rapidly, and others more difficult [55].

A key to this debate is the notion of what represents an objective correct answer—that is, a response that signifies accuracy in a decision task that participants understand. The structure of the choice-from-array studies described above instead used intended response as a dependent measure, which is decided by the researchers and tied to culturally based assumptions of emotional signalling. Statistical models that calculate the probability of accuracy in a task assume that accuracy is operationalized appropriately. The null hypothesis must constitute a legitimate theoretical baseline (i.e. be objectively inaccurate). Gendron *et al*. [49] claimed that their task contained a correct answer, but they conflated ‘correct’ with ‘intended’, that is, intended by the experiment's designer. Again, participants heard a story read to them followed by two non-verbal vocalizations, and were asked to choose the sound that best corresponded to the story. In a provided example, ‘Someone is suddenly faced with a dangerous animal and feels very scared’, the authors assumed the correct response would be the fear vocalization, which in their case was a scream. But many of the other ‘incorrect’ vocalizations might constitute a legitimate (i.e. correct or corresponding) response to the situation. For example, depending on the animal, perhaps screaming is not appropriate—many indigenous societies have quite specific cultural knowledge regarding how to engage with animals. Some Himba subjects might believe that not making any sound at all could be the most appropriate response. If so, the fact that Himba subjects did not pair a scream with the dangerous animal context could be a valid and justifiable response, and would not demonstrate that Himba lack a concept of fear or an understanding of the emotional significance of a scream.

As an example of research in which participants were asked to make judgements for which there were objectively correct answers, not formulated or constructed by the experimenters, consider a study that examined vocal emotion recognition in the Shuar, an Amazonian hunter–horticulturalist society [56]. Vocal emotions were elicited in speakers by having them look at pictured emotional faces and then emulate the emotions in the face with their voice. Simple sentences were created in English, and actors spoke these sentences using the affective prosody associated with different emotional faces. The judgement task was simple: subjects were presented two faces, played a single vocal recording and then asked to indicate which face the speaker was trying to imitate. Every vocal stimulus was produced while looking at a specific face, and one of these faces was always included as a choice in the judgement task. No emotion terms were used in the task, but participants were introduced to the pictures with emotion labels. One of the two pictures constituted a correct answer, since which face the speaker was trying to imitate was a matter of fact. Using this method, subjects were able to identify the vocalizations produced for anger, sadness, happiness and fear faces better than chance, and their errors patterned as expected given form–function relationships between different emotional categories [39]. Other vocal emotion studies using similar paradigms incorporating an actual correct answer have also demonstrated high cross-cultural consistencies in responses (e.g. [57–59]).

To be clear: these experiments, like most, were not theory-free. In the study of vocal emotion recognition in Shuar adults, the stimuli were constructed using facial expression exemplars that were selected in order to test specific hypotheses about which emotions can be distinguished through the voice. However, the correctness of the answers had nothing to do with the experimenters' choices or theories. Speakers were either imitating the face in question, or they were not. By contrast, Gendron *et al*. [49] used vocal emotion stimuli that even within-culture (American) judges could not reliably identify better than 70% of the time. The task not only failed to contain a correct answer, but the intended answers were often not easily recognized. Triumph was recognized only 5% of the time, and sensory pleasure barely above 40%. Their ‘nonword’ stimuli even contained English informal lexical exclamations that might not be understood across cultures (e.g. ‘Woohoo’, ‘Ewww’). Moreover, Himba participants provided answers that were inappropriate to the task 69% of the time, revealing they did not really understand what was asked of them. When accuracy is being measured—as presumed in both the task design and statistical analysis that Gendron *et al*. employed—it is crucial not only that there be a correct answer, but also that ambiguity in what subjects are being asked to do is minimized [60].

The debate over universality in emotions is currently focused largely on disagreements between (i) early proposals mapping basic categories such as anger, happiness and fear to distinct neurocognitive action patterns (e.g. [61]), and (ii) constructionist accounts of emotions that describe highly fluid and dynamically unfolding emotion concepts subject to the forces of language and culture (e.g. [62]). But both approaches are theoretically approaching emotion signalling at the wrong level of analysis to discover evolved design features. Selection has shaped our emotion programmes, and all associated multimodal signals (including vocalizations), to solve a large suite of adaptive social communication problems. On this view, each domain of emotional signalling will have its own computational problem space which can include many constraints on any given modality in both production and perception, as well as complex multimodal integration that operates in tandem during highly variable social contexts. For emotional signalling systems to evolve, some universality is necessary, making at least some aspects of the constructionist viewpoint untenable. A fundamental question, therefore, is not whether universality exists, but rather, what form it takes—not something that can be determined *a priori*. In order to detect whatever universals might exist in human communication systems, it is important that we use methodology that is suited to the task.

## 5. Infant-directed speech

Infant-directed (ID) speech is one of the earliest voice modulation phenomena to be examined across languages and cultures. Ferguson [63] described ID speech in six languages, including Arabic, Marathi, Comanche, Gilyak, American English and Spanish. Many common features were noted across these languages, as were language-specific features. In terms of strictly vocal (i.e. acoustic) features, this analysis was not extensive, but Ferguson noted some phonological phenomena across most of the six languages, such as simplification of consonant clusters and various consonant replacements. Interestingly, he pointed out that there seemed to be differences across cultures in attitudes about public displays of ‘babytalk’, which relates to the idea that pragmatic variations across societies can drive differences in other dimensions. In a later analysis, Ferguson [64] described ID modifications in ‘speech register’ across 15 languages and over 20 societies, although this category can include linguistic features such as vocabulary and syntax, not just voice modulation.

Later cross-cultural work began investigating acoustic features of ID speech more specifically. A handful of early studies examined ID speech in languages other than English, including German (e.g. [65,66]) and Mandarin Chinese (e.g. [67,68]). Even across Indo-European languages, there are reasons to expect some variation in the way adults use prosody to communicate effective meanings. Fernald *et al*. [69] noted that stress-timed languages such as English and German use *f*_o_ prominence for signalling emphasis but syllable-timed languages like French use duration to mark stress, and use *f*_o_ prominence to indicate word boundaries. And closely related languages like Italian use prosodic signals relatively less for word order, and instead rely differentially on lexical cues. These variations in the prosodic structure, even within a fairly narrow linguistic group, could have impacts on how ID speech manifests itself across languages.

Fernald *et al*. [69] examined prosodic features in ID speech across five languages: French, Italian, German, Japanese and English (British and American). Using 10 speakers (five mothers and five fathers) in each of the six language groups, ID speech was recorded in their home environments and basic acoustic properties were analysed. There was a high degree of consistency across languages, with ID speech having relatively higher *f*_o_, *f*_o_ variability, shorter utterances and longer pauses than adult-directed (AD) speech. But there were variations as well, including different patterns across mothers and fathers, and differences across languages. Notably, American parents showed the most extreme prosodic modifications, which is consistent with research in other domains of vocal research. This work was one of the first to point out the problem of developing theoretical accounts of vocal behaviour based largely on research in one group, in this case, American English speakers—a group that happens to be on the extreme end of a continuum, a common pattern for WEIRD populations [11].

These early studies not only revealed apparent near universality of particular acoustic features such as greater average pitch and pitch variability, but also that prosodic contours varied systematically across different kinds of contexts. Fernald [70] showed how distinct communicative intentions such as approvals, prohibitives, attention and comforting afforded different contours. For example, prohibitive utterances tend to have an abrupt, high-energy onset, often lowered pitch and short, burst-like utterances. Conversely, comforting vocalizations, such as attempting to regulate the arousal of an infant who is crying and upset, will have a gentle sound characterized by low amplitude, lilting rhythms and high pitch. ID singing also follows these forms, with playsongs and lullabies having prosodic features that map onto context-specific intentions geared towards regulating arousal and attention [71]. Naturally, there can be overlap in these categories as well, such as attention-getting features being prominent in prohibitives or comforting features being included in approvals. This is what a form–function account predicts: prosodic forms across similar interactive contexts should converge as a function of communicative intent in speakers.

ID speech is designed to be perceptually salient, with distinctive acoustic features notable enough that infants prefer it in a foreign language over AD speech in their native language [72]. Similar recognizable features manifest cross-culturally in ID songs (such as lullabies) as well [73,74], and compared with other types of songs, infants relax when hearing lullabies in a foreign language [75]. Two recent massive cross-cultural studies on ID vocal communication have provided by far the best evidence to date that there are perceptible, structural regularities in acoustic features of ID speech and song across disparate cultural and language groups [76,77]. Moser *et al*. [77] created a corpus of 1614 vocalizations, including ID and AD speech and singing, from over 400 vocalizers in 21 societies. Machine classifiers revealed acoustic distinctions across the four categories of vocalizations, many revolving around *f*_o_ dimensions, and these distinctions explained almost half the variability in judgements made by over 13 000 naive participants. Consistent with much earlier work, infant-directedness was associated with greater average pitch and pitch variability, though a host of other variables related to voice quality also seem important (e.g. energy in the second formant of ID speech, which could be relevant for vowel category acquisition).

Interestingly, ID song was more reliably judged as being directed towards infants than ID speech, which in some cases was not consistently judged as ID relative to AD speech. Across the corpus, ID speech was clearly recognized, but participants in small-scale societies did not always systematically detect it, raising questions regarding how these acoustic forms might vary across cultural groups. One possibility is that the freedom afforded to vocalizers in generating the tokens could have created variability that confounded some judgements as different types of ID speech (e.g. prohibitives versus approvals) will have specific acoustic features associated with them. ID song, alternatively, might manifest itself more consistently across quite different cultures, and thus be more recognizable owing to its potentially more stereotyped, ritualized sound characteristics. Earlier work from the same research group found that lullabies are a particularly robust category of music that is widely produced and recognized across cultures [74].

Judges can distinguish between basic intention categories across highly disparate cultures (e.g. [59,78]). While listeners can make these judgements in forced-choice paradigms (with the same caveats as described earlier), there are also clear variations in how adults and older children modulate their voices when speaking to young infants, driven by cultural and linguistic factors. One basic dimension of variation is just the simple likelihood that people talk to babies in the first place. Early scepticism regarding universals in ID speech was often driven by this measure—scholars can potentially ignore specific modifications in ID speech because of apparent low base rates for its occurrence (e.g. [79]). There are some notable cultural differences in how much adults talk to babies, with some societies doing it at relatively high rates (e.g. North American [80]), and others doing it rather infrequently (e.g. Tsimane of Bolivia [81]).

Besides variation in the rates of ID speech occurrence across cultures, there are also varying strategies in when and how to produce it. Evidence suggests not only that WEIRD parents produce ID speech more often, but that some do it in more extreme ways than others. Broesch & Bryant [82] found that US and non-Western (Fijian and Kenyan) mothers both produced ID speech using higher *f*_o_ and *f*_o_ variation (s.d.) than when producing AD speech, but US mothers increased *f*_o_ to a greater extent than their non-Western counterparts. Interestingly, when mothers' education was controlled in the analysis, the cultural difference disappeared. In an analysis of fathers’ ID speech, including US, Canadian and Ni-Vanuatu (small-scale rural islanders) men, ID speech was always modified relative to AD speech, but in different ways [83]. Fathers from Ni-Vanuatu tended to use higher *f*_o_ when speaking to their infants, but their speech rate did not change. Conversely, fathers from the USA and Canada did not alter their *f*_o_ but tended to use slowed ID speech relative to AD speech. These results showed that speakers can adopt different strategies when modifying their speech to infants, and these variations likely relate to many factors, including cultural conventions in its use, communicative functions of its types and individual differences in communicative style.

There are many approaches that speakers can take to achieve largely the same goals, and these strategies do not always need to include vocalizations. This is true of all types of spoken communication. As in adult spoken language, there are many ways to achieve the same outcome—linguistic diversity reveals incredible variation. ID speech is no exception. A form–function account, however, predicts that certain strategies should be most common in specific domains. For example, given the specific acoustic function of loud noises to rapidly interrupt dangerous or otherwise undesirable behaviour in babies, we should expect this to vary less than, for example, the ways to encourage behaviour in infants. Few communicative tactics will interrupt behaviour in infants (or adults) as effectively as an abrupt yell. And as one might expect, this kind of vocalization is widely recognized, and likely manifests itself universally for that purpose [59,70]. But communicating approval can be done effectively through different modalities, such as facial signals, body gestures, voices and basic language. Thus, we should expect more variability for this intention category across cultures.

In some ways, ID speech research has been able to avoid the methodological pitfall of failing to use an objectively correct criterion in dependent measures. For one, perception studies examining whether presented vocal recordings were directed to an infant or another adult measure accuracy in that exact dimension (i.e. recordings generally were actually ID or AD). On the production side, researchers can quantify precise acoustic features in vocal recordings and statistically demonstrate direct relationships between acoustic measurements and judgement patterns of those tokens. Consequently, cross-cultural research has been able to establish reliable (and increasingly undeniable) patterns of universality in ID speech, and cultural variations are easier to detect and interpret. But questions of function are more difficult to address, especially as ID speech appears to be multifunctional, with early effects being related to affective communication, and later functions being potentially connected to different aspects of language learning [70,84,85].

The growing literature on universal acoustic forms in ID speech suggests shared functions across cultures, but almost no work has explored this issue carefully. In fact, there is relatively little direct evidence for proposed adaptive benefits of ID speech more generally. Studies done primarily in English speakers and other WEIRD societies have revealed various effects, including increased brain activation in response to ID speech relative to AD speech, and enhanced language learning (for reviews, see [86,87]). The paucity of work on functional effects of ID speech is likely due in part to difficulties associated with longitudinal measurement over developmental time. But even relatively simple studies examining the direct impacts of ID speech on immediate behaviour are lacking. For example, we should expect that infants across disparate societies should respond similarly to basic acoustic phenomena such as abrupt, loud vocalizations interrupting behaviour, and modulated, musical sounds resulting in increased relaxation in moments of distress (e.g. crying). The form–function approach affords specific predictions of how ID speech sounds should affect infants' behaviour, and these effects should transcend cultural boundaries.

## 6. Conclusion

Vocal communication is central to the social life of humans and many other species. As in the behavioural sciences more generally, voice research has focused primarily on WEIRD participants despite a great need to explore the clear relevant variation that exists across people from different linguistic and cultural groups. Recently, however, there has been a positive trend of including participants from a wide diversity of populations. But prior views on universals rooted in the concept of innateness have limited our vision both theoretically and methodologically. Species-typical traits can vary dramatically owing to flexibility in reaction norms and plasticity in function [88]. Thus, traits in vocal production and perception systems develop features best explicable as distributions rather than fixed categories, which will be revealed in cross-cultural analyses [8]. Within the domain of vocal signalling, distinct adaptive problems select for particular design features, and these features can result in more or less variation as a function of input such as language, culture and context. We must refine our theoretical expectations to fit the specific research problems we face, and not interpret deviations from typical patterns as a chance to refute a broad theoretical construct, such as universality.

An important methodological consideration for cross-cultural researchers is the use of proper dependent measures for evaluating consistencies in behaviour across different societies. Attempts to assess the extent to which different groups share properties in any kind of psychological process or trait should rely on a measurement system that functions equivalently across the cultural boundaries. Assuming that materials are translated properly—establishing functional and cultural equivalence—tasks should be measuring the same thing for all participants. In the case of a decision task where a given response is scored as ‘correct’, the response should generally adhere to objective criteria for what actually constitutes a correct response. Reliance on language-based categories becomes immediately suspect by this standard, and researchers presuming themselves to be studying objective judgements are sometimes inadvertently studying opinions, misunderstandings or even just noise. As described earlier, this problem is especially troublesome for emotion perception research as the phenomena of interest are not inherently linguistic, and behaviour patterns are not subject to a single correct alternative (e.g. which vocalization is appropriate in a given emotional scenario).

Many cross-cultural vocal researchers have managed to largely avoid this predicament by relying on objective acoustic measures that can be implemented across cultures, and judgements of objective properties of speakers such as speaker size and strength or the intended target of a vocalizer (e.g. ID or AD). In such research, there are relatively more consistencies found across disparate cultural groups, as opposed to studies that measure opinions such as attractiveness or social status. That said, there is certainly value in measuring variations in opinion-based, subjective judgements. For example, cross-cultural research on attractiveness in small-scale societies has revealed tremendous variation that provides important insights into mating psychology, significantly refining earlier evolutionary-based assumptions (e.g. [89,90]). But researchers must recognize the subjective aspects of their measures, if they exist. These kinds of data likely track individual differences well, but I argue here that they typically do not provide evidence against universality in the mechanisms that drive those judgements.

The future of cross-cultural vocal communication research is bright. Technology is increasingly affording massive data collection efforts, including sophisticated, high-quality voice recordings and interdisciplinary collaborations allowing the establishment of multidimensional databases. Our recently enhanced ability to conduct this kind of research must be matched by a refinement in our cross-cultural methodologies and theoretical frameworks. An evolutionary perspective that integrates human voice research with the vast literature on non-human vocal communication, as well as the cognitive science of human social behaviour, will best afford the important recognition of both the deep homologies of vocal control and perception, and human uniqueness in how we communicate with our voices.

## Endnote

1 In evolutionary biology, signals are communication adaptations designed to affect the behaviour of other organisms, and are generally complemented by receiver adaptations, resulting in mutual benefits for senders and receivers on average. Cues, in this context, are any acts or structures *not* designed to affect the behaviour of other organisms, but reveal information to receivers incidentally. Receivers can have evolved responses to cues, but cues do not evolve to have effects on receivers (see [18]).

## Data accessibility

This article has no additional data.
